# A steady state analysis indicates that negative feedback regulation of PTP1B by Akt elicits bistability in insulin-stimulated GLUT4 translocation

**DOI:** 10.1186/1742-4682-1-2

**Published:** 2004-08-03

**Authors:** Lopamudra Giri, Vivek K Mutalik, KV Venkatesh

**Affiliations:** 1 Department of Chemical Engineering and School of Biosciences and Bioengineering, Indian Institute of Technology Bombay, Powai, Mumbai-400076, India

**Keywords:** Insulin signaling pathway, GLUT4, Translocation, Enzyme cascade, Feedback loops, Bistable switch

## Abstract

**Background:**

The phenomenon of switch-like response to graded input signal is the theme involved in various signaling pathways in living systems. Positive feedback loops or double negative feedback loops embedded with nonlinearity exhibit these switch-like bistable responses. Such feedback regulations exist in insulin signaling pathway as well.

**Methods:**

In the current manuscript, a steady state analysis of the metabolic insulin-signaling pathway is presented. The threshold concentration of insulin required for glucose transporter GLUT4 translocation was studied with variation in system parameters and component concentrations. The dose response curves of GLUT4 translocation at various concentration of insulin obtained by steady state analysis were quantified in-terms of half saturation constant.

**Results:**

We show that, insulin-stimulated GLUT4 translocation can operate as a bistable switch, which ensures that GLUT4 settles between two discrete, but mutually exclusive stable steady states. The threshold concentration of insulin required for GLUT4 translocation changes with variation in system parameters and component concentrations, thus providing insights into possible pathological conditions.

**Conclusion:**

A steady state analysis indicates that negative feedback regulation of phosphatase PTP1B by Akt elicits bistability in insulin-stimulated GLUT4 translocation. The threshold concentration of insulin required for GLUT4 translocation and the corresponding bistable response at different system parameters and component concentrations was compared with reported experimental observations on specific defects in regulation of the system.

## Background

In living systems, extracellular information is processed through signal transduction machinery to appropriately regulate cellular function. This information processing machinery is made up of a complex web of enzyme cascades, allosteric interactions and feedback loops. Depending on their regulatory design these signaling networks elicit diverse responses, but display many common operating principles. A recurring theme in signaling systems is switch-like responses to graded or transient input signal. Various mechanisms are known to generate such all-or-none responses [1]. Bistability is one such system level property, in which, the system switches between two discrete stable steady states without being able to rest in an intermediate state. Bistable systems exhibit hysteresis wherein, the value of input stimulus required for system transition from one state to another is quite different from the value required for reverse transition. Both computational and experimental analyses have shown that bistability plays a significant role in cellular differentiation and cell cycle progressions [2-5], production of biochemical memory [6], microbial metabolic systems [7], lateral signal propagation [8] and protein translocations [9]. Existence of bistability in cellular regulation has been attributed to nonlinearity embedded in positive feedback loop or double negative feedback loop [10]. Here, we present steady state simulation results of metabolic insulin signaling pathway comprising of positive feedback loops and show that this system can convert graded inputs into switch-like bistable output response.

Insulin is the most potent anabolic peptide hormone known that elicits myriad biological responses by specifically binding to insulin receptor and simultaneously stimulating multiple signaling pathways to regulate growth, differentiation and metabolism. Insulin maintains glucose homeostasis by stimulating the uptake, utilization and storage of glucose in muscle and adipose tissue, and inhibits hepatic glucose production [11]. Defects in any of the pathway components lead to disturbance in growth, differentiation, and in the homeostasis of glucose and lipid levels. This leads to disease conditions such as type 2 diabetes, hypertension, obesity and a cluster of abnormalities characterized by insulin resistance or deficiency. In such a condition, normal circulating concentration of insulin is insufficient to elicit appropriate response [12,13]. Studies over the last century have identified the major insulin signaling components involved in the regulation of glucose uptake into cells and its various defects in diseased states.

**Figure 1:**
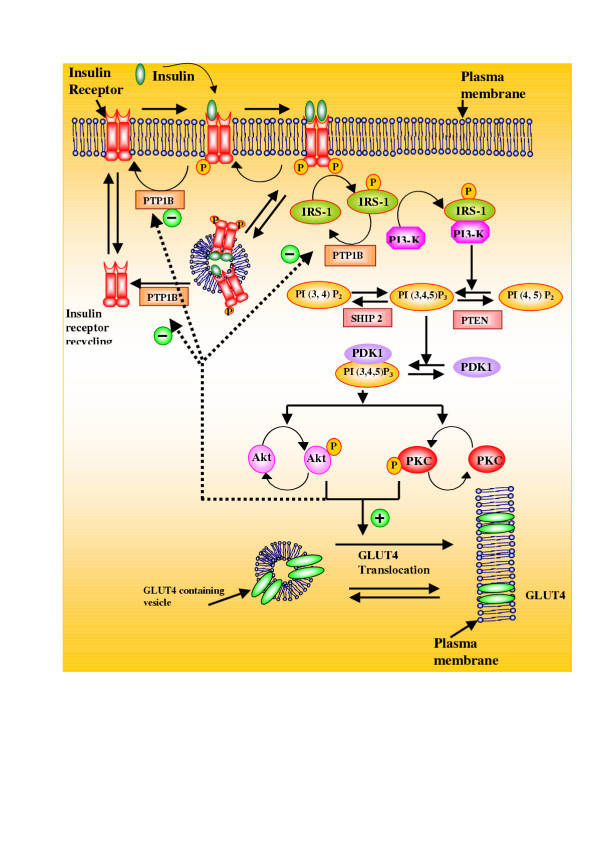
**Simplified representation of molecular mechanism involved in insulin signaling pathway that regulates glucose transporter (GLUT4) translocation to cell membrane. **Some of the details like, other isoforms of insulin receptor substrate and multiphosphorylation of insulin receptor substrate are not shown here. Nomenclature: GLUT4: Glucose-transporter isoform 4; IRS-1: Insulin receptor substrate-1; PI3K: Phosphatidylinositol-3-kinase; PI (3, 4, 5) P3: Phosphatidylinositol (PI)-3, 4, 5-tiphosphate; PDK1: phosphosinsositide-dependent kinase 1; Akt: Protein kinase Akt or protein kinase B (PKB); PKC: Protein kinase C-ς; PTP1B: Protein tyrosine phosphatase 1B; PTEN: 3' lipid phosphatase; SHIP2: 5' lipid phosphatase; Detailed description of signaling events are given in the methods section. Letter 'P' indicates phosphorylated species.

A wide family of glucose-transporter proteins localized in the plasma membrane, facilitate uptake of glucose from the blood into tissues. Among different isoforms, only glucose transporter isoform-4 (GLUT4) is specifically expressed to promote glucose uptake in insulin sensitive tissues, viz. muscle and adipose, and in response to insulin, GLUT4 gets translocated to the plasma membrane from intracellular vesicles [14]. The biological action of insulin is initiated by binding to the tyrosine kinase receptor and its subsequent activation. The activated tyrosine kinase receptor undergoes autophosphorylation and catalyzes the phosphorylation of several intracellular substrates including the insulin-receptor substrate (IRS) proteins (Fig. 1). The activated IRS isoform-1 protein further activates downstream components to elicit translocation of GLUT4 [11]. There are several downstream kinases like PI-3 kinase, Akt (or protein kinase B) and protein kinase C-ζ (PKC-ζ) demonstrated to be potentially capable of phosphorylating upstream proteins like IRS-1 and tyrosine phosphatase 1B (PTP1B) thus serving as negative and positive feedback loops respectively [15]. Other than feedback loops, crosstalk between multitudes of signal transduction pathways have also been reported, thus making the insulin-signaling pathway a highly intricate network [11].

Although studies on various cell lines, transgenic and knock-out mice, have helped to uncover and characterize the different components involved in insulin signaling pathway, there are many voids in our understanding of the precise molecular mechanisms of signal transduction and cellular effects of insulin [16,17]. The major hurdles are complexity of insulin signaling pathway and technical problems like experimental methodology employed for system level quantification. For example, depending upon different techniques employed, quantification of GLUT4 translocation in response to insulin binding yielded different results in the same cell type [18]. Recent technical developments however have helped in studying the localization and translocation of signaling proteins and overall quantification of signaling processes in single cells has been possible [19]. In such a scenario, it is pertinent to ask questions regarding the design principles involved in intracellular regulation. For example, what does a particular regulatory structure accomplish and how does it help in exhibiting different physiological responses. Based on available experimental data, computational and mathematical analysis can answer some of these questions and possibly propose new experiments and hypotheses. Earlier mathematical modeling studies of insulin signaling pathways have focused on subsystems of the pathway, like insulin receptor binding kinetics [20,21], receptor recycling [22] and GLUT4 translocation [23-25]. Recently a comprehensive dynamic model of metabolic insulin signaling pathway was presented, which involved most of the known signaling components [26]. Although the model correlated well with the published experiment data, authors did not discuss the system level regulatory design of insulin signaling system.

In the present work, we have developed a steady state model of insulin signaling to generate dose response curves for fractional translocation of GLUT4 to varying input insulin stimuli. One of the main objectives was to investigate the effect of inherent signaling structure made up of phosphorylation cycles, allosteric interactions and feedback loops on the system level response of insulin on GLUT4 translocation. Furthermore, we were interested in examining whether the regulatory design consisting of positive feedback loops in insulin signaling pathway exhibits bistable response. We solved the steady state equations for the entire metabolic insulin pathway including the positive feedback loops numerically, and found that GLUT4 gets translocated to the plasma membrane in an all-or-none manner in response to a varying concentration of input insulin stimuli. We show that GLUT4 translocation switches between the on-state and off-state and exhibits hysteresis in its response to increasing and decreasing input insulin concentration. This input-output relationship was then studied at various concentration of signaling components and system parameters in order to monitor the range over which this response persisted. We discuss these results by comparing with the known specific defects in regulation of the system (insulin dependent diseases) that lead to improper glucose uptake into the cell.

## Methods

**Figure 2:**
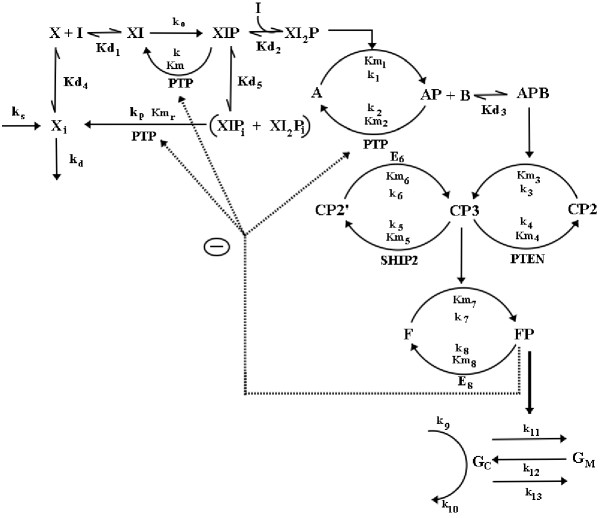
**Schematic representation of metabolic Insulin signaling pathway used for the steady state analysis. **Nomenclature: I, Insulin; X, unbound surface insulin receptor; XI, unphosphorylated once-bound surface receptor; XIP, phosphorylated once-bound surface receptor; XI_2_P, phosphorylated twice-bound surface receptor; X_i _represents intracellular receptor pool; XIP_i _and XI_2_P_i _are internalized form of XIP and XI_2_P; phosphatase PTP catalyzes the dephosphorylation of AP, XIP, XIP_i _and XI_2_P_i_. A, unphosphorylated IRS-1; AP, phosphorylated IRS-1; B, inactive PI3-kinase; APB, phosphorylated IRS-1 and PI3-kinase complex; CP3, lipid PI[3,4,5]P_3_; CP2, lipid PI[4,5]P_2_; CP2', lipid PI[3,4]P_2_; phosphatase SHIP2 catalyzes dephosphorylation of CP3 to form CP2', phosphatase PTEN catalyzes dephosphorylation of CP3 to form CP2; F, inactive Akt and PKC-ς; FP, phosphorylated Akt and PKC-ς; E_8 _dephosphorylates FP; E_6 _phosphorylates CP2' to form CP3; FP activates GLUT4 from intracellular location to plasma membrane. G_C _and G_M _represent GLUT4 in cytoplasm and on plasma membrane respectively. Kd_1 _to Kd_3 _are dissociation constants; Kd_4 _and Kd_5 _are distribution coefficients; Km_r_, Km, Km_1_to Km_8 _are Michaelis-Menten constants; k, k_p_, k_d_, k_s_, k_0_, k_1 _to k_13 _are reaction rates as shown in the figure.

Figure 1 shows a simplified representation of molecular mechanisms involved in insulin signaling pathway. The metabolic insulin-signaling pathway used for the steady state simulation in the present work is shown in Fig. 2. This schematic representation is a compilation of various interactions in insulin pathway which have been very well reviewed [11-27]. We have used the framework of Goldbeter and Koshland [28] to model the insulin system at steady state and accordingly an equivalent rate constant and Michaelis-Menten constant nomenclature scheme is applied. The detailed list of the steady state equations for covalent modification cycles, equilibrium relationships for allosteric interactions, mass balance equations for total species and parameters used in the simulations are provided in Appendix. All component enzyme concentrations are represented with respect to whole cell volume. Most of the kinetic/equilibrium constants are taken from the literature. In this analysis, the reactants like ATP and PPi concentrations are assumed to be constant. In the following paragraphs we present the system considered and assumptions made during the analysis.

Insulin initiates its biological action by interacting with the insulin receptor, which belongs to a superfamily of tyrosine kinase receptors. On binding to the first insulin molecule, the receptor gets auto-phosphorylated and is dephosphorylated by phosphatase PTP1B [12]. The phosphorylated insulin receptor can either bind with another insulin molecule or undergoes dissociation. Binding of the second insulin molecule does not affect the phosphorylation state of the receptor. Here we have assumed that the concentration of unbound phosphorylated receptor is negligible. Thus, phosphorylated receptors can exist as species bound to either singly or doubly bound molecules of insulin. Insulin bound phosphorylated receptor rapidly gets internalized into the endosomal apparatus of the cell before it gets dephosphorylated by PTP1B and incorporated into intracellular receptor pool [29]. However recent studies indicate that, PTP1B might interact with insulin receptor directly and deactivate it without internalization [30]. We have assumed that, the membrane bound phosphorylated insulin-receptor and its internalized form, both get dephosphorylated by PTP1B. The rate equation for intracellular receptor at steady state is represented as



where k_p _is rate constant and Km_r _is Michaelis-Menten constant for dephosphorylation of internalized insulin receptors XIP_i _and XI_2_P_i_. The term k_d _is first order degradation rate constant and k_s _is zero order synthesis rate constant of intracellular receptor X_i_. The receptor exocytosis and endocytosis are assumed to be at quasi-equilibrium because of their faster time scales than the synthesis and degradation of receptors [26].

The phosphorylated active receptors further catalyze phosphorylation of several intracellular substrates including the IRS proteins, GAB-1, Shc and c-Cab1 [16]. Among these, IRS-1 protein is known to participate in the regulation of GLUT4 translocation. In the present study we have assumed that, at steady state the twice-bound phosphorylated receptor catalyses the phosphorylation of IRS-1 protein while neglecting the activation of GAB-1, Shc, c-Cab1.

The phosphorylated active IRS-1 further binds and activates PI3 kinase and this association is assumed to occur with a stoichiometry of 1:1. Activated PI3 kinase further phosphorylates phosphatidylinositol-(4,5)-bisphosphate (PI-4,5-P2) to form phosphatidylinositol -3,4,5-triphosphate, (PIP3). The dephosphorylation of PIP3 to form PI-4,5-P2 is catalyzed by phosphatase PTEN, whereas, PIP3 is dephosphorylated to form PI-3,4-P2 by phosphatase SHIP2. Active PIP3 then is known to interact allosterically with phosphosinsositide-dependent kinase 1 (PDK1) and which in turn appears to phosphorylate kinase Akt (or protein kinase B) and protein kinase C-ζ (PKC-ζ) [11]. However, as the interaction due to PDK1 is unclear, active PIP3 is assumed to play a role in phosphorylation of Akt and PKC-ζ. Since the parameters affecting the modification-demodification of Akt and PKC-ζ are considered to be similar, their modification is represented as a single enzyme cascade (Fig. 2).

The downstream elements of Akt and PKC-ζ, which effect GLUT4 translocation, are also unknown [11-13]. Therefore, we have assumed that phosphorylated Akt and PKC-ζ directly activate the GLUT4 translocation to the plasma membrane. In the basal state, GLUT4 slowly recycles between the plasma membrane and intracellular vesicular compartment. The phosphorylated Akt and PKC-ζ favor GLUT4 translocation (exocytosis) to the plasma membrane and thus increase glucose uptake as a response to insulin binding to the receptor [14]. Here, total GLUT4 (G_t_) is assumed to be sum of GLUT4 concentration in the cytosol (G_C_) and on the membrane (G_M_). The rate equation for GLUT4 species in cytoplasm at steady state is represented by,



where, k_9 _is the basal zero order synthesis rate of GLUT4, k_10 _is basal first order degradation rate, k_11 _is the insulin-activated GLUT4 exocytosis, k_12 _and k_13 _are basal first order rate of exocytosis and endocytosis, respectively. As assumed by Sedaghat, *et al*. [26], the basal equilibrium distribution of cell surface GLUT4 and GLUT4 in the intracellular pool are taken as 4% and 96%.

The insulin signaling pathway has been shown to consist of multiple feedback loops [15]. Active Akt is known to phosphorylate and thereby negatively regulate the upstream phosphatase PTP1B. This phosphorylation impairs the ability of PTP1B to dephosphorylate insulin receptor and IRS-1 by 25% [31]. This represents overall positive feedback loop as Akt inhibits signal attenuation enzyme PTP1B. The resulting circuit also represents a double negative feedback loop, in which phosphorylated protein negatively regulate the phosphatase that dephosphorylates it. To incorporate these feedback loops we assumed that active Akt affects the total active PTP1B enzyme and thus inhibits the dephosphorylation of the receptor and IRS-1. The feedback effect of Akt on PTP1B was incorporated by following relationship



where, [PTP]_max _is maximum PTP1B concentration, PTP_t _is the total active PTP1B concentration after incorporating the effects of feedback, AktP represents the phosphorylated Akt concentration influencing the PTPase activity, and k_f _represents the half saturation constant quantifying feedback. The value of k_f _was estimated based on the assumption that 25% of PTP1B is inactivated by total AktP [31]. Thus, k_f _is appropriately calculated so that the first term [k_f _/[k_f _+ AktP]] is equal to 0.75. In absence of feedback effects, PTP_t _equals PTP_max_.

The set of equations given in 'appendix' and in 'methods' section were solved numerically using fsolve program of Matlab (The MathWorks Inc. USA). The accuracy of the simulation was verified by numerically checking the mass balance of all species. The steady state modeling of entire insulin signaling was evaluated including the feedback loops and estimating the fractions of GLUT4 translocated to the plasma membrane for a particular concentration of insulin. Thus, the overall action of insulin on GLUT4 translocation is quantified as,



where, *f *is fractional GLUT4 on plasma membrane, G_M _is GLUT4 concentration on plasma membrane and G_t _is total GLUT4 concentration with respect to whole cell volume.

## Results

### Bistability in GLUT4 translocation to plasma membrane

**Figure 3:**
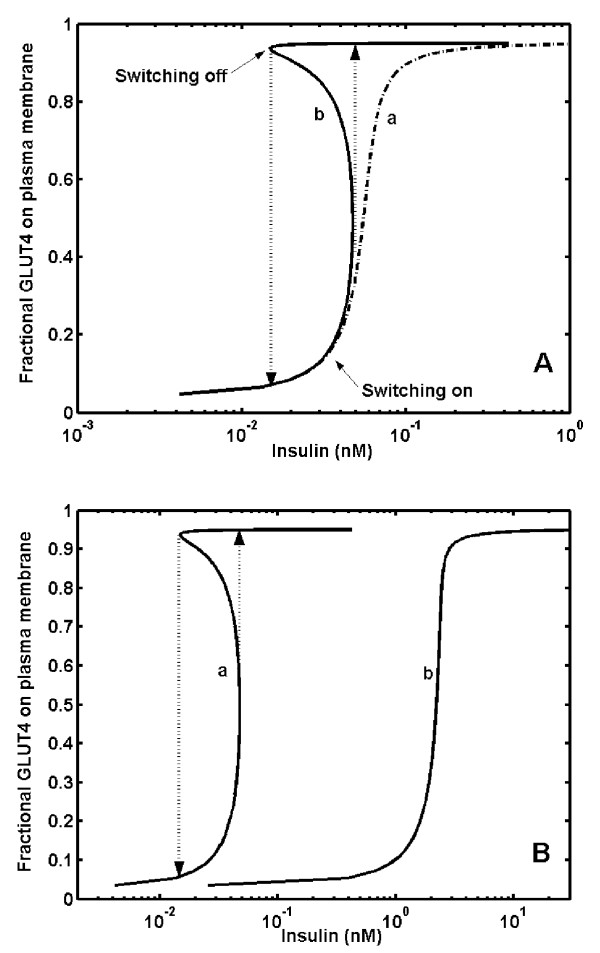
**Hysteresis and bistability in insulin-stimulated GLUT4 translocation. *A***. Dose response curve of insulin stimulated fractional GLUT4 on plasma membrane. Curve 'a' is sigmoidal dose response curve [~Hill coefficient of 3.1] obtained in absence of feedback loop. Curve 'b' represents hysteresis in insulin-stimulated fractional GLUT4 on plasma membrane in presence of feedback loop which impairs the ability of PTPase by 25%. Arrows indicate the switching-on [up arrow] and switching-off [down arrow] GLUT4 translocation. ***B***. A simulated type 2 diabetic condition represented by dose response curve of insulin-stimulated fractional GLUT4 on plasma membrane at higher phosphatase PTP1B concentration. Curve 'a' is typical bistable response obtained in presence of positive feedback loops [PTP1B conc. 0.039 nM]. Curve 'b' represents dose response curve when PTPase concentration was increased by 3 fold [PTP1B conc. 0.098 nM]. A 3-fold increase in the PTPase concentration increased the half-maximal concentration by 100 fold and the response looses bistability.

Fig. 3A shows the predicted dose response curve of steady state fraction of GLUT4 bound to the plasma membrane at different concentrations of insulin. The predicted dose response curve indicates that, there are three steady states exist between 0.01 nM and 0.05 nM of insulin for GLUT4 translocation (curve b, Fig 3A). Out of these three steady states, GLUT4 gets distributed between two discrete stable steady states, either at plasma membrane or in the cytosol without settling in an intermediate unstable state, thus showing a typical hysteresis response. Due to hysteresis, the dose response curve appears to split and we obtain two distinct half-maximal concentrations (K_0.5_, insulin concentration required for 50% of GLUT4 to reside on the plasma membrane). This represents two threshold concentrations of insulin required for GLUT4 translocation switching on (GLUT4 translocation to plasma membrane at 0.05 nM) and switching off (GLUT4 translocation from to plasma membrane at 0.01 nM).

The observed hysteresis is characteristic of a bistable response obtained due to negative feedback regulation of upstream signal attenuation enzyme PTP1B by downstream kinase Akt. Experimental data available in the literature indicates a subsensitive response of insulin, requiring ~130 fold change in insulin concentration for the maximal GLUT4 translocation to plasma membrane [32]. Our results show an ultrasensitive response in insulin-stimulated GLUT4 translocation due to bistability (~4-fold change in insulin concentration); however, the half saturation values match with that of experimental data. The response was ultrasensitive (Hill coefficient ~3.1) and not bistable in absence of feedback loops (curve a, Fig 3A).

### Effect of system component concentration on GLUT4 translocation

To examine the influence of pathological conditions arising due to variations in protein expression levels on final output response of insulin, we varied the concentration of individual signaling components IRS-1, PI3K, lipids, PKC-ζ, Akt and phosphatases, PTP1B, PTEN and SHIP2 over a wide range. For each case, the dose response curve of fractional GLUT4 on the plasma membrane at various insulin concentrations was obtained and the response was quantified in-terms of half saturation constant. To illustrate this, we consider a case of increase in PTP1B concentration. Fig. 3B shows the dose response curves for insulin stimulated GLUT4 translocation at plasma membrane at two different concentrations of PTP1B. At high PTP1B concentration, the bistable dose response curve becomes monostable (but, still ultrasensitive) and shifts to the right. This indicates a nullifying effect of negative feedback regulation on PTP1B by Akt and higher requirement of insulin for maximal translocation of GLUT4. Thus, in Fig 3B curve 'a' and curve 'b' can be characterized by two and one half saturation values respectively.

**Figure 4:**
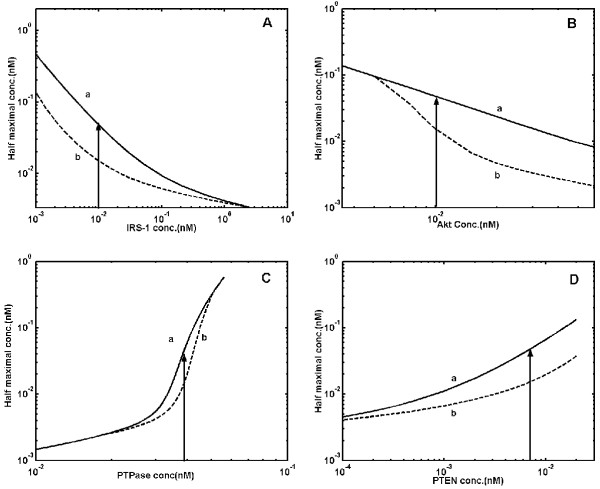
**Half-maximal concentration of insulin required for 50% GLUT4 translocation at elevated levels of signaling components. **Curve 'a' shows half maximal concentration of insulin required to switch-on GLUT4 translocation. Curve 'b' shows half maximal concentration of insulin required to switch-off GLUT4 translocation. Arrow indicates physiological concentration of particular signaling components. ***A***. Half saturation constant at various concentration of IRS-1. Simulated results indicate increased insulin sensitivity when IRS-1 overexpressed. ***B***. Half saturation constant at various concentration of Akt. Simulated results indicate increased insulin sensitivity when Akt overexpressed and loss of bistability when Akt concentration decreased below 0.01 nM. ***C***. Half saturation constant at various concentration of PTP1B. Simulated results indicate decreased insulin sensitivity when PTP1B overexpressed. ***D***. Half saturation constant at various concentration of PTEN. Simulated results indicate decreased insulin sensitivity when PTEN overexpressed.

Fig. 4A and 4B show the distinct half saturation constant values obtained for switching-on and switching-off of GLUT4 translocation at various concentrations of IRS-1 and Akt respectively. Such an increase or decrease in the half-maximal concentration of insulin characterizes the decrease and increase in insulin sensitivity found in diseased conditions. The threshold concentration of insulin required for switching-on GLUT4 translocation decreases with increase in IRS-1 concentration. This implies that, increase in IRS-1 concentration amplifies the input signal and beyond a certain concentration of IRS-1 [~3 nM], the system looses bistability. Similar results were obtained for variations in lipid, PI3K and insulin receptor concentration (results not shown). GLUT4 translocation at various concentrations of Akt shows that the system becomes monostable when Akt concentration is decreased. However, the degree of bistability (i.e., difference between half maximal concentrations for switch-on and off) increases with increase in Akt concentration and furthermore, the threshold value to activate GLUT4 translocation decreases.

To study the effect of signal attenuation enzymes such as phosphatases on the output response, the concentrations of PTP1B, PTEN and SHIP2 were altered over a wide range, keeping other parameters constant. Fig. 4C and 4D show the influence of variation in concentrations of PTP1B and PTEN on half saturation constant of insulin. Increase in PTP1B and PTEN concentration results in a drastic increase in the threshold concentration of insulin required to switch-on or switch-off GLUT4 translocation. This illustrates that more insulin than physiological concentration is required at higher phosphatase (PTP1B or PTEN) concentrations to translocate GLUT4 from cytoplasm to plasma membrane. For example, around 16-fold change in the insulin concentration is observed for a 1.5-fold increase in PTP1B concentration from 0.039 nM to 0.06 nM. The system looses bistability beyond a narrow range of PTP1B concentration between 0.02 nM to 0.05 nM. Thus, the response of GLUT4 translocation to insulin is particularly sensitive to PTP1B concentration.

### Influence of feedback on GLUT4 translocation

**Figure 5:**
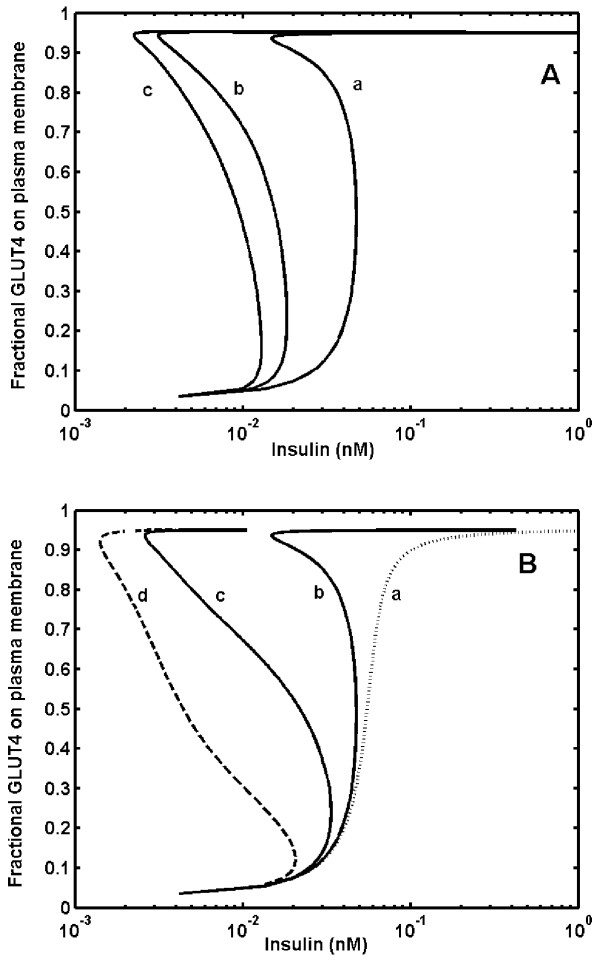
**Influence of feedback effects on bistable insulin-stimulated GLUT4 translocation. *A***. Bistable response with increase in the concentration of Akt representing increased non-linearity due to zero order ultrasensitivity. Dose response curves obtained at different Akt concentrations: Curve 'a', 0.01 nM; Curve 'b', 0.03 nM; Curve 'c', 0.05 nM. ***B***. Influence of percentage of feedback effects on dose response curve of insulin-stimulated GLUT4 translocation. The percentage feedback represents the percentage by which the dephosphorylation ability of PTP1B is impaired. Dose response curves obtained: Curve 'a' in absence of feedback; Curve 'b' 25% feedback effect; Curve 'c' 67% feedback effect; Curve 'd' 90% feedback effect.

The feedback effect of active Akt on PTP1B was studied by increasing the Akt concentration (Fig. 5A) and by changing the percentage feedback at a fixed Akt concentration (Fig. 5B). As shown in Fig. 5A, increase in Akt concentration amplifies the signal by maintaining bistable response. Similarly, by increasing the percentage feedback at a fixed Akt concentration, (Fig. 5B) the degree of bistability dramatically increased, while not influencing the threshold concentration required for switching-on the response. The bistable response was not observed when percentage feedback was smaller or in absence of feedback loops. In absence of receptor internalization, 65% inhibition of PTP1B by Akt was required to display a bistable response, whereas, inclusion of receptor internalization demonstrated bistability even at 25% inhibition of PTP1B.

The steady state analysis of metabolic insulin-signaling pathway demonstrated signal amplification as signal propagates down the cascades. The amount of insulin required for 50% activation of insulin receptor, IRS-1, PIP3, Akt, PKC-ζ and GLUT4 was estimated to decrease in presence or absence of feedback loops (results not shown).

### Effect of system parameter values on GLUT4 translocation

**Figure 6:**
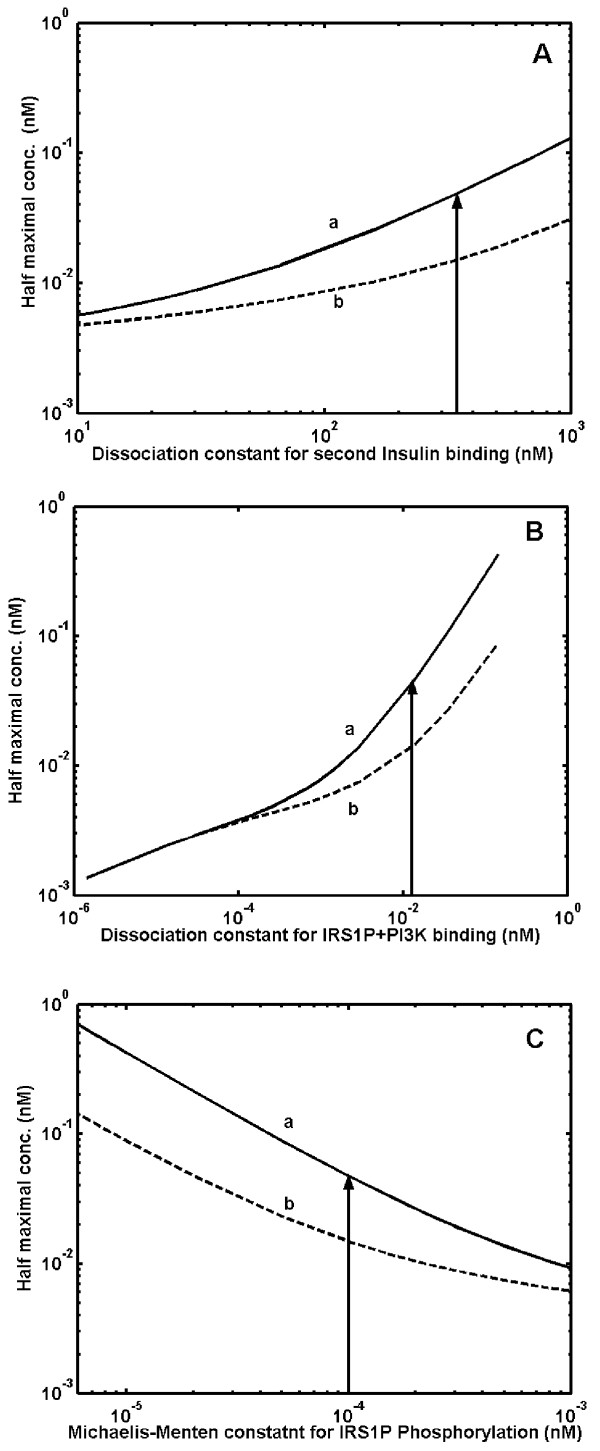
**Effect of key system parameter values on GLUT4 translocation. **Curve 'a' shows half maximal concentration of insulin required to switch-on GLUT4 translocation. Curve 'b' shows half maximal concentration of insulin required to switch-off GLUT4 translocation. Arrow indicates parameter used in the simulation. ***A***. Half maximal concentration of insulin required for GLUT4 translocation at different values of dissociation constant [Kd_2_] for binding of second molecule of insulin to phosphorylated insulin bound receptor. Simulated results indicate decreased insulin sensitivity when Kd_2 _increased. ***B***. Half maximal concentration of insulin required for GLUT4 translocation at different values of dissociation constant [Kd_3_] for binding of phosphorylated IRS-1 to PI3K species. Simulated results indicate decreased insulin sensitivity when Kd_3 _increased. ***C***. Half maximal concentration of insulin required for GLUT4 translocation at different values of Michaelis-Menten constant [Km_2_] for dephosphorylation of phosphorylated IRS1 by PTP1B. Simulated results indicate decreased insulin sensitivity when Michaelis-Menten constant [Km_2_] was decreased due to increased affinity with dephosphorylating enzyme.

In addition to genetic variation at the protein expression levels in diseased conditions, mutational changes can also alter the system parameters and thereby modify the final output response. To examine the influence of system parameter values on insulin-stimulated GLUT4 translocation, we have analyzed the performance of insulin signaling pathway to variations in key parameter values such as, dissociation constant and Michaelis-Menten constant. Increase in dissociation constant quantifying the interaction between insulin-receptor and phosphorylated IRS-1-PI3K shows an increase in the half saturation constant indicating higher requirement of insulin over the physiological concentration (Fig. 6A and 6B). The system becomes monostable at very low values of dissociation constants. Similarly, decrease in the Michaelis-Menten constant of the dephosphorylation cycles, also increases the half saturation constant, thus decreasing the insulin sensitivity (Fig. 6C). Simulation results indicate that, the alterations in binding constant of allosteric interactions and Michaelis-Menten constants in modification-demodification cycles in the insulin-signaling pathway can result in insulin resistance or diabetes.

## Discussion

In this work we have demonstrated that, the dose response curves of fractional GLUT4 concentration on plasma membrane at various concentration of insulin exhibit hysteresis-a property of bistable systems. The analysis of bistable response in presence of feedback loops was done at varying concentration of signaling components and system parameters in physiological range. The overall response of insulin demonstrated signal amplification as the signal propagates down the cascade, thus requiring less insulin for GLUT4 translocation. The insulin sensitivity increased by increasing the concentration of proteins that amplify the insulin action and decreasing the concentration of proteins that attenuate insulin-signaling pathway. This indicates that the bistability and the half saturation constant are dependent on the component concentrations and system parameters.

It is known that defects in insulin signaling pathway leads to pathological conditions like diabetes, wherein normal or elevated levels of insulin produces impaired biological response. This characteristic decrease or increase in insulin sensitivity is mainly attributed to post-receptor defects including mutational changes in protein expression levels or other parameters like dissociation constants and Michaelis-Menten constants [13,33]. Numerous experimental studies like targeted deletions/mutations of signaling components have yielded insights about the disease states. In the present work, to study the influence of pathological conditions on final output response of insulin, the concentration of individual signaling components was varied over a wide range, by keeping other parameters constant. The predicted results are consistent with various reported experimental observations and thus validate our steady state model. (*i*) Decreased concentration of phosphorylated insulin receptor and IRS-1 are observed in muscle from morbidly obese subjects [34] and those with diabetes [35]. This decreased phosphorylation can be either due to decrease in protein concentration itself or impaired phosphorylation event. (*ii*) Increase in the level and activity of several tyrosine phosphatases (PTP1B) was found to be associated with insulin resistance and reduced insulin sensitivity [12,13,33,36]. (*iii*) Overexpression of PI3K and its downstream targets Akt and PKC in cell culture models increased GLUT4 translocation [12]. (i*v*) Decrease in the association of PI3K with phosphorylated IRS-1 and subsequent activation appears to be a characteristic abnormality in type 2 diabetes and obesity [13,33-35]. (*v*) Single gene knockout experiments have shown that, mice with PTP1B knockout [37], mice with SHIP2 knockout [38] and targeted deletion of PTEN in murine lever [39], all results in hypersensitivity towards insulin. In the present work, though we have not done *in-silico *perturbation analysis by deleting a particular protein, we have changed the concentration of specific protein over wide range to bring about the similar effect of deficiency. Thus, our simulation results show that the insulin sensitivity dramatically increased when we decreased the concentration of phosphatases like PTP1B, PTEN and SHIP2.

Increase in the concentration of Akt, makes the signal amplification increased along with slight increase in the degree of bistability. This effect is brought about by the enhanced nonlinearity in the feedback loop due to zero order ultrasensitivity [28] imposed by increasing the concentration of Akt or percentage feedback. At high Akt concentration (or when overexpressed), the system can respond in constitutively active mode or might also function as a memory module. That is, once insulin switches on the system, the high Akt concentration or percentage feedback by itself can keep the switch on even after insulin is removed. This may be the reason for the experimental observation of insulin independent GLUT4 translocation to plasma membrane when Akt is overexpressed [12,40]. This insulin independent translocation of GLUT4 is thought to be due to activation of PI3K independent pathway or by amplification of residual signal. Our analysis indicates that the inherent feedback structure present in the insulin-signaling pathway by itself can induce this phenomenon.

### Does GLUT4 translocation show a bistable response to insulin in-vivo?

Bistability has been shown to be the outcome of regulatory structure with feedback loops and non-linearity in the system [41]. The non-linearity in the system is brought about by an ultrasensitive response typically obtained through enzyme cascades. Such ultrasensitive responses exhibit steep dose response curves with Hill coefficient greater than one [1]. The cascade systems use energy for their operation and are optimally operated under zero order sensitivity (i.e., cascades operating under saturating interconvertable enzymes) requiring minimum energy [42,43]. Presence of feedback loops may further optimize the requirement of energy. Enzyme cascades and multiple positive feedback loops are observed in insulin-signaling pathway. Experimental results have shown that the dose response curve of insulin-stimulated glucose uptake is subsensitive with a Hill coefficient of about 0.8 [calculated from ref. [32]]. Thus the question arises as to what may be the significance of the cascade signaling system with positive feedback loops in insulin signaling pathway. The reason for this discrepancy may be because, the experimental data represents a profile of glucose uptake in ensemble of cells [32], and furthermore, glucose uptake may not be proportional to the amount of GLUT4 on cell surface [18].

Recently, bistability has been experimentally demonstrated in many cellular regulation systems [10]. Experiments on cellular differentiation and cell-cycle progression have shown that, to demonstrate bistability it is essential to measure the amount of input required to switch-on the system from a low activity state to a high activity state, and amount of input required to hold-on the system in high activity state [3-5]. Reynolds *et al*. [8], have shown experimentally that, the reaction network of PTPase inhibition by activated epidermal growth factor receptor (EGFR, a receptor tyrosine kinase, RTK) through reactive oxygen species, can generate highly amplified and switch like bistable response to a threshold concentration of ligand stimulus. In this system, PTPase is a negative regulator of active RTK and thus, PTPase inhibition by reactive oxygen species serves as a positive feedback loop.

Our simulation results indicate that similar bistable response can be obtained in insulin-stimulated GLUT4 translocation because of the positive feedback loops (inhibitory action of Akt on PTP1B). Though experimental verification of this property is awaited, there are indications that insulin signaling pathway possesses many requisite components to exhibit bistable response. The simulation results presented here showed that, the ultrasensitivity in absence of feedback effects and the regulatory structure of feedback loops are main reasons for a bistable response. Other than the positive feedback loops considered in the present work, Insulin signaling system is also known to contain many feedback loops which have not been entirely characterized [15]. One possible reason for having so many interlocking positive feedback and negative feedback loops may be to cause oscillations in GLUT4 translocation or to make the bistability of GLUT4 translocation – more robust.

Recently, it has been shown that insulin stimulation in a variety of cell types elicit a rapid production of hydrogen peroxide and which reversibly inhibit PTP1B enhancing propagation of the early insulin signal [44]. This regulatory mechanism was also found to be essential for PI3K mediated Akt activation, GLUT4 translocation to plasma membrane and insulin-stimulated glucose uptake [45]. However, unlike EGFR system [8] existence of bistable behavior is yet to be shown in insulin signaling system. In insulin signaling pathway other than GLUT4, proteins like Akt and PKC get translocated to plasma membrane and such inter-compartmental translocations can also exhibit switch like bistable response [9].

It is becoming clear that quantification studies have to be performed in single cell rather than cell populations [19]. This is true especially while addressing the system level questions like ultrasensitivity, bistability and oscillations [4-7,46]. Recently, this was also found to be of immense value in case of insulin signaling pathway to uncover the PIP3 activation mode [47]. Simultaneous measurement of PIP3 production and GLUT4 insertion in individual 3T3L1 adipocytes suggested that a threshold for PIP3 signals exists. Below this threshold, GLUT4 transporters are minimally inserted into the plasma membrane and their insertion increases once this threshold is overcome. In summary, it is essential to show through experiments that, the stimulus-response curve for insulin-stimulated GLUT4 translocation exhibits hysteresis, – a distinctive splitting in stimulus response curve. There should be a particular concentration of insulin, which is not sufficient to translocate GLUT4 to plasma membrane, but is sufficient to maintain GLUT4 on plasma membrane.

What may be the significance of such a bistable behavior in GLUT4 translocation? Though there is no obvious answer to this question yet, it is known that, bistability can maintain a biological response even when the input stimulus is brief and the high activity level is maintained only as long as the system requires. Insulin binding to its cell surface receptor is rapidly followed by internalization of insulin-receptor complex. This internalization of receptor has been implicated in receptor down regulation, attenuation of insulin sensitivity and insulin clearance from the circulation [12,13]. Thus a brief input stimulation should be sufficient to balance the translocation of GLUT4 to plasma membrane and its reversal depending on glucose concentration. Additionally, due to small absolute stimulus concentrations, the probability of noise occurrence is high. A bistable response having distinct threshold concentration to switch-on and switch-off offers advantage in handling noise.

In cellular regulation, different structural motifs such as enzyme cascades, feedforward control and multiple feedback loops yield complex regulatory networks. These are shown to be responsible for exhibiting system level properties including bistability and robustness [10,48,49]. Other than multiple feedback loops, structural regulatory motifs like multisite covalent modification cycles have been shown to induce bistability [50]. The interconnections between these regulatory motifs at the system level may elicit a multistable response to particular stimuli, which have to be theoretically uncovered and demonstrated through experiments.

## Abbreviations used

GLUT4: Glucose-transporter isoform 4;

IRS: Insulin-receptor substrate;

PI3K: Phosphatidylinositol-3-kinase;

PIP3: Phosphatidylinositol (PI)-3,4,5-tiphosphate (PI-3,4,5-P3);

Akt: Protein kinase Akt or protein kinase B (PKB);

PKC: Protein kinase C;

PTP1B: Protein tyrosine phosphatase 1B;

PTEN: 3' lipid phosphatase;

SHIP2: 5' lipid phosphatase;

## Competing Interests

None declared.

## Author's Contributions

LG, VKM and KVV conceived and designed the experiments. LG performed the experiments. LG, VKM and KVV analyzed the data. VKM and KVV conceptualize the manuscript. All authors have read and approved the final manuscript

## Appendix

**Equilibrium relationships, rate equations, mass balance equations and model parameters used for simulation of metabolic insulin signaling system at steady state (refer Fig. **2** for nomenclature and interaction details). **Equations were solved simultaneously, for evaluating fractional GLUT4 translocation at a particular insulin concentration, using fsolve function of Matlab (The MathWorks Inc. USA). Most of the values of model parameters for steady state analysis are taken from Sedaghat *et al*. [26]. Nomenclature, parameter values are:

### Component concentrations

I_t_, total insulin concentration varied over a wide range; X_t_, total receptor = 0.003 nM; A_t_, total IRS-1= 0.01 nM, B_t_, total PI3-Kinase = 0.5 nM, PTEN_t_, total PTEN= 0.007 nM; CP2_t_, total lipid = 0.01 nM; SHIP2_t_, total SHIP2 = 0.001 nM; F_t_, total Akt+PKC-ξ = 0.02 nM, PTP_max_, total PTP1B= 0.039 nM; G_t_, total GLUT4 = 9 nM; E_6t_, total E_6 _= 0.001 nM; E_8t_, total E_8 _= 0.001 nM;

### Rate constants

k_0 _= 2500 min^-1^; k = 0.2 min^-1^; k_1_= 4.16 min^-1^; k_2 _= 1.4 min^-1^; k_3 _= 50 min^-1 ^(assumed); k_4 _= 42.1 min^-1^; k_5 _= 2.8 min^-1^; k_6 _= 3 min^-1^; k_7 _= 20 min^-1 ^(assumed); k_8 _= 6.9 min^-1^; k_9 _= 0.11 min^-1^; k_10 _= 0.0012 min^-1^; k_11 _= 3.47 min^-1 ^(assumed); k_12 _= 6.96*10^-3 ^min^-1^; k_13 _= 0.17 min^-1^; k_p _= 0.461 min^-1 ^; k_d _= 1.67 × 10^-18 ^min^-1 ^; k_s _= 1.67*10^-25 ^nM min ^-1^;

### Dissociation constants

Kd_1_= 3.33 nM; Kd_2 _= 333.3 nM; Kd_3 _= 0.014 nM;

### Distribution coefficients

Kd_4 _= 9 nM; Kd_5 _= 0.1 nM;

### Michaelis-Menten constants

Km_r_, Km_1 _to Km_8 _= 10^-4 ^nM

The total molar balances for different species are given below.

*I*_*t *_= *I *+ *XI *+ *XIP *+ 2*XI*_2_*P *+ *PTP*.*XIP *+ *A*.*XI*_2_*P *+ *XIP*_*i *_+ 2*XI*_2_*P*_*i *_+ *PTP*.*XIP*_*i *_+ *PTP*.*XI*_2_*P*_*i *_    [A1]

*X*_*t *_= *X *+ *X*_i _+ *XI *+ *XIP *+ *XI*_2_*P *+ *XIP*_i _+ *XI*_2_*P*_i _+ *PTP*.*XIP *+ *A*.*XI*_2_*P *+ *PTP*.*XIP*_i _+ *PTP*.*XI*_2_*P*_i _    [A2]

*A*_*t *_= *A *+ *AP *+ *APB *+ *A*.*XI*_2_*P *+ *PTP*.*AP *+ *APB*.*CP2 *    [A3]

*CP*2_*t *_= *CP*2 + *CP*3 + *CP*2' + *APB*.*CP*2 + *PTEN*.*CP*3 + *SHIP*2.*CP*3 + *SHIP*2.*CP*3 + *E*_6_.*CP*2' + *F*.*CP*3     [A4]

*F*_*t *_= *F *+ *FP *+*CP*3*F *+ *E*_8_.*FP *    [A5]

*SHIP*2_*t *_= *SHIP*2 + *SHIP*2.*CP*3     [A6]

*PTEN*_*t *_= *PTEN *+ *PTEN*.*CP*3     [A7]

*PTP*_*t *_= *PTP *+ *PTP*.*XIP *+ *PTP*.*AP *+ *PTP*.*XIP*_*i *_+ *PTP*.*XI*_2_*P*_*i *_    [A8]

*B*_*t *_= *B *+ *APB *+ *CP*2.*APB *    [A9]

*E*_6*t *_= *E*_6 _+ *E*_6_.*CP*2'     [A10]

*E*_8*t *_= *E*_8 _+ *E*_8_.*FP *    [A11]

*G*_*t *_= *G*_*M *_+ *G*_*C *_    [A12]

Equilibrium relationships











Rate expression with pseudo-steady state representation of complexes for modification-demodification cycles

Receptor autophosphorylation and dephosphorylation cycle



IRS-1 phosphorylation and dephosphorylation cycle



Phosphorylation and Dephosphorylation of PI-4,5-P2, PI-3,4-P2 and PIP3





Phosphorylation and Dephosphorylation of Akt + PKC


